# Higher migraine risk in healthcare professionals than in general population: a nationwide population-based cohort study in Taiwan

**DOI:** 10.1186/s10194-015-0585-6

**Published:** 2015-12-03

**Authors:** Wan-Yin Kuo, Chien-Cheng Huang, Shih-Feng Weng, Hung-Jung Lin, Shih-Bin Su, Jhi-Joung Wang, How-Ran Guo, Chien-Chin Hsu

**Affiliations:** Department of Emergency Medicine, Chi-Mei Medical Center, 901 Zhonghua Road, Yongkang District, Tainan City, 710 Taiwan; Department of Environmental and Occupational Health, College of Medicine, National Cheng Kung University, Tainan, Taiwan; Department of Child Care and Education, Southern Taiwan University of Science and Technology, Tainan, Taiwan; Department of Occupational Medicine, Chi-Mei Medical Center, Tainan, Taiwan; Department of Geriatrics and Gerontology, Chi-Mei Medical Center, Tainan, Taiwan; Department of Healthcare Administration and Medical Informatics, Kaohsiung Medical University, Kaohsiung, Taiwan; Department of Biotechnology, Southern Taiwan University of Science and Technology, Tainan, Taiwan; Department of Emergency Medicine, Taipei Medical University, Taipei, Taiwan; Department of Leisure, Recreation and Tourism Management, Southern Taiwan University of Science and Technology, Tainan, Taiwan; Department of Medical Research, Chi Mei Medical Center, Liouying, Tainan, Taiwan; Departments of Medical Research, Chi-Mei Medical Center, Tainan, Taiwan; Department of Occupational and Environmental Medicine, National Cheng Kung University Hospital, Tainan, Taiwan

**Keywords:** Healthcare professionals, Migraine, Nurse, Physician, Specialty

## Abstract

**Background:**

High stress levels and shift work probably trigger migraine in healthcare professionals (HCPs). However, the migraine risk differences between HCPs and the general population is unknown.

**Methods:**

This nationwide population-based cohort study used Taiwan’s National Health Insurance Research Database. Physicians (50,226), nurses (122,357), and other HCPs (pharmacists, technicians, dietitians, rehabilitation therapists, social workers, etc.) (45,736) were enrolled for the study cohort, and randomly selected non-HCPs (218,319) were enrolled for the comparison cohort. Conditional logistical regression analysis was used to compare the migraine risks. Comparisons between HCPs and between physician specialties were also done.

**Results:**

Physicians, nurses, and other HCPs had higher migraine risks than did the general population (adjusted odds ratio [AOR]: 1.672; 95 % confidence interval [CI]: 1.468–1.905, AOR: 1.621; 95 % CI: 1.532–1.714, and AOR: 1.254; 95 % CI: 1.124–1.399, respectively) after stroke, hypertension, epilepsy, anxiety, depression, and insomnia had been adjusted for. Nurses and physicians had higher migraine risks than did other HCPs (AOR: 1.303; 95 % CI: 1.206–1.408, and AOR: 1.193; 95 % CI: 1.069–1.332, respectively). Obstetricians and gynecologists had a lower migraine risk than did other physician specialists (AOR: 0.550; 95 % CI: 0.323–0.937).

**Conclusion:**

HCPs in Taiwan had a higher migraine risk than did the general population. Heavy workloads, emotional stress, and rotating night shift sleep disturbances appear to be the most important risk factors. These findings should provide an important reference for promoting occupational health in HCPs in Taiwan.

## Background

Migraine is one of the most prevalent neurological disorders: it affects up to 12 % of the general population [1–5] and is seventh highest specific cause of disability worldwide [6]. Migraine was the best studied of the headache disorders, from all aspects including epidemiologically [6]. Because it is recurrent, migraine is a potentially debilitating disease that reduces work, daily, and school activity [1, 4, 6–9]. In 2001, the World Health Organization recognized migraine as an important public health concern and listed it as one of the leading causes of disability in the world [10].

Work stress, one of the environmental factors, is believed to be a significant factor in migraine [11]. Healthcare professionals (HCPs) have stressful jobs, are frequently on rotating work shifts, undergo emotional stress, and work long hours every day because of their job requirements [12–14]. In Canada, nearly half (45 %) of the HCPs reported highly stressful work days [15]. Physicians and nurses were especially stressed [15].

The risk for migraine in HCPs is not well understood. One study [16] reported that approximately 29 % of nurses in Taiwan had migraine, and another [17] that approximately 15 % of nurses in northern China did. Although other studies have also reported the prevalence of migraine in HCPs, most were hospital-based and had small sample sizes. Furthermore, the migraine risk in other HCPs compared with that in the general population, and HCPs in general and physician specialties has never been clarified. Therefore, we did a nationwide population-based cohort study in Taiwan to examine these questions. We hypothesized that migraine risk is higher in HCPs, and especially in physicians and nurses, because of their greater job stress.

## Methods

### Data source

**Fig. 1:**
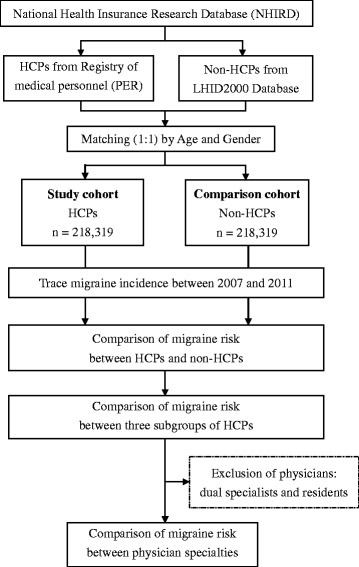
The flowchart of this study. *HCP* health care professional, *LHID* Longitudinal Health Insurance Database

Taiwan’s National Health Insurance (NHI) program, established in 1995, currently covers more than 99 % of the country’s legal residents [18]. The NHI Research Database (NHIRD), which contains registration files and original claim data from reimbursement, is one of the largest administrative healthcare databases in the world [18]. It provides patient identification number, gender, age, date of visit, length of hospitalization, prescribed medication, and diagnoses using the International Classification of Diseases, Ninth Revision, Clinical Modification (ICD-9-CM) [18]. Registration files of HCPs obtained from the Registry of medical personnel (PER) of NHIRD include residence area, hospital level, type of employment, specialty, date of HCP license, and encrypted identification number (Fig. 1). Non-HCPs were recruited from the Longitudinal Health Insurance Database 2000 (LHID2000), a data subset of the NHIRD that contains all claims data of one million (4.34 % of the total population) random beneficiaries. There is no significant difference of the characteristics between LHID2000 and NHIRD [18]. NHI covers all the expenses of migraine, stroke, hypertension, epilepsy, anxiety, depression, and insomnia treatments.

### Ethics statement

This study was done according to the ethic principles of the Declaration of Helsinki and approved by the Chi-Mei Medical Center Institutional Review Board (IRB). Because the dataset used in this study consisted of deidentified information for academic research, the IRB waived the need for informed consent from the enrolled patients. Nevertheless, patient rights and welfare were not affected.

### Selection of study cohort (HCPs) and comparison cohort (non-HCPs)

The data of the HCPs were acquired from all of the registered records the Registry of Medical Personnel (PER) in 2009. HCPs were classified into three subgroups: physicians, nurses, and other HCPs (pharmacists, technicians, dietitians, rehabilitation therapists, social workers, etc.) (Fig. 1). An identical number of non-HCPs were randomly selected from the LHID 2000 as the comparison cohort. Each HCP was matched to one non-HCP by age and gender. In addition to demographic information, we examined migraine-associated comorbidities: stroke (ICD-9 code 434.91), hypertension (ICD-9 codes 401–405), epilepsy (ICD-9 code 345.10), anxiety (ICD-9 code 300.00), depression (ICD-9 code 300.4), and insomnia (ICD-9 code 780.52). These six comorbidities were counted if the enrollee had the diagnosis in 3 or more ambulatory care claims before January 1, 2009.

### Comparison between HCPs and non-HCPs

We traced all the enrollee’s medical records to compare the migraine risk between HCPs and non-HCPs from 2007 to 2011. The ICD-9 code of 346.90 indicates a diagnosis of migraine.

### Comparison between HCPs and physician specialists

We compared the migraine risk between three subgroups of HCPs (physicians vs. other HCPs, and nurses vs. other HCPs) (Fig. 1). Physicians were also categorized by specialty—internal medicine, surgery, obstetrics and gynecology [Obs/Gyn], pediatrics, family medicine, emergency medicine, etc.—and then compared. Physicians with dual specialties (e.g., board certified for surgery and emergency medicine) were excluded because it was difficult to assign them to an individual specialty. Residents were also excluded because they usually had rotating training specialties and insufficient experience.

### Statistical analyses

Differences in baseline demographic characteristics and baseline comorbidities between the groups were analyzed using Student’s *t* test (continuous variables) and the Pearson *χ*^2^ test (categorical variables). The migraine risk between the HCPs and comparisons was compared using conditional logistic regression analysis. The probable confounding factors of stroke, hypertension, epilepsy, anxiety, depression, and insomnia were adjusted for. The migraine risks between HCPs and physician specialists were compared using unconditional logistic regression analysis. SAS 9.3.1 for Windows (SAS Institute, Cary, NC, USA) was used for all analyses. Significance was set at *P* < 0.05 (two-tailed).

## Results

The enrollees in this study included 50,226 physicians, 122,357 nurses, 45,736 other HCPs and an identical number of age- and gender-matched non-HCPs. The mean ages of the three subgroups of HCPs were 44.42 ± 12.15 (physicians), 33.55 ± 8.76 (nurses), and 38.37 ± 10.88 (other HCPs) (Table 1). Most physicians (81.56 %) were men, and most nurses (98.97 %) and other HCPs (61.87 %) were women. All three subgroups of HCPs had a significantly higher risk of anxiety and insomnia than did non-HCPs. They also had a higher risk of hypertension, but the difference in nurses did not reach statistical significance. In contrast, all three subgroups of HCPs had significantly lower risks of epilepsy and depression than didnon-HCPs. They also had a lower risk of stroke, but the difference in nurses did not reach statistical significance.

**Table 1:** Demographic Characteristics, baseline comorbidities, and residence location for health care professionals (HCPs) and non-HCPs in Taiwan

	Physicians (*n* = 50,226)	Non-HCPs (*n* = 50,226)	*P*	Nurses (*n* = 122,357)	Non-HCPs (*n* = 122,357)	*P*	Other HCPs (*n* = 45,736)	Non-HCPs (*n* = 45,736)	*P*
Age (years)									
0–34	12,477 (24.84)	12,477 (24.84)	>0.999	76,955 (62.89)	76,955 (62.89)	>0.999	20,355 (44.51)	20,355 (44.51)	>0.999
35–59	22,001 (43.80)	22,001 (43.80)		38,096 (31.14)	38,096 (31.14)		17,383 (38.07)	17,383 (38.07)	
≥ 60	15,748 (31.35)	15,748 (31.35)		7306 (5.97)	7306 (5.97)		7998 (17.49)	7998 (17.49)	
Age (years)	44.42 ± 12.15	44.42 ± 12.15	>0.999	33.55 ± 8.76	33.55 ± 8.76	>0.999	38.37 ± 10.88	38.37 ± 10.88	>0.999
Gender			>0.999			>0.999			>0.999
Female	9263 (18.44)	9263 (18.44)		121,096 (98.97)	121,096 (98.97)		28,297 (61.87)	28,297 (61.87)	
Male	40,963 (81.56)	40,963 (81.56)		1261 (1.03)	1261 (1.03)		17,439 (38.13)	17,439 (38.13)	
Baseline comorbidity									
Stroke			<0.0001			0.3152			<0.0001
Yes	1133 (2.26)	1657 (3.30)		701 (0.57)	739 (0.60)		550 (1.20)	705 (1.54)	
No	49,093 (97.74)	48,569 (96.70)		121,656 (99.43)	121,618 (99.40)		45,186 (98.80)	45,031 (98.46)	
Hypertension			<0.0001			0.0671			<0.0001
Yes	9742 (19.40)	8375 (16.67)		5554 (4.54)	5367 (4.39)		4913 (10.74)	4412 (9.65)	
No	40,484 (80.60)	41,851 (83.33)		116,803 (95.46)	116,990 (95.61)		40,823 (89.29)	41,324 (90.35)	
Epilepsy			<0.0001			<0.0001			<0.0001
Yes	71 (0.14)	234 (0.47)		193 (0.16)	300 (0.25)		66 (0.14)	157 (0.34)	
No	50,155 (99.86)	49,992 (99.53)		122,164 (99.84)	122,057 (99.75)		45,670 (99.86)	45,579 (99.66)	
Anxiety			<0.0001			<0.0001			<0.0001
Yes	3298 (6.57)	2926 (5.83)		8446 (6.90)	6969 (5.70)		3463 (7.57)	2751 (6.01)	
No	46,928 (93.43)	47,300 (94.17)		113,911 (93.10)	115,388 (94.30)		42,273 (92.43)	42,985 (93.99)	
Depression			<0.0001			<0.0001			<0.0001
Yes	1056 (2.10)	1424 (2.84)		3691 (3.02)	4094 (3.35)		1150 (2.51)	1420 (3.10)	
No	49,170 (97.90)	48,802 (97.16)		118,666 (96.98)	118,263 (96.65)		44,586 (97.49)	44,316 (96.90)	
Insomnia			<0.0001			<0.0001			<0.0001
Yes	4743 (9.44)	2658 (5.29)		10,954 (8.95)	6216 (5.08)		4256 (9.31)	2447 (5.35)	
No	45,483 (90.56)	47,568 (94.71)		111,403 (91.05)	116,141 (94.92)		41,480 (90.69)	43,289 (94.65)	
Residence location			<0.0001			<0.0001			<0.0001
North	24,396 (48.57)	26,256 (52.33)		57,346 (46.87)	65,157 (55.75)		21,577 (47.18)	24,752 (54.17)	
Central	10,204 (20.32)	8877 (17.69)		22,008 (17.99)	21,520 (17.60)		8954 (19.58)	7980 (17.47)	
South	14,468 (28.81)	14,044 (27.99)		39,447 (32.24)	30,434 (24.90)		14,153 (30.94)	12,089 (26.46)	
East	1158 (2.31)	994 (1.98)		3556 (2.91)	2135 (1.75)		1052 (2.30)	869 (1.90)	

Data are number (%) or mean ± SD

The cumulative incidence rates of migraine were 1.51 % in physicians, 3.28 % in nurses, and 1.96 % in other HCPs in this 5-year study (Table 2). Physicians, nurses, and other HCPs had a higher migraine risk than did non-HCPs after adjusting for age, gender, stroke, hypertension, epilepsy, anxiety, depression, and insomnia (adjusted odds ratio [AOR]: 1.672; 95 % confidence interval [CI]: 1.468–1.905; AOR: 1.621; 95 % CI: 1.532–1.714; and AOR: 1.254; 95 % CI: 1.124–1.399, respectively). Compared with other HCPs, both physicians (AOR: 1.193; 95 % CI: 1.069–1.332) and nurses (AOR: 1.303; 95 % CI: 1.206–1.408) had a higher migraine risk (Table 3).

**Table 2:** Comparison of migraine risk between healthcare professionals (HCPs) and non-HCPs by conditional logistic regression analysis

	Number (%)	Crude OR (95 % CI)	AOR (95 % CI)^a^
Physicians (*n* = 50,226)	759 (1.51)	1.572 (1.402–1.763)**	1.672 (1.468–1.905)**
Non-HCPs (*n* = 50,226)	485 (0.97)	1.00	1.00
Nurses (*n* = 122,357)	4008 (3.28)	1.634 (1.553–1.719)**	1.621 (1.532–1.714)**
Non-HCPs (*n* = 122,357)	2483 (2.03)	1.00	1.00
Other HCPs (*n* = 45,736)	897 (1.96)	1.236 (1.119–1.364)**	1.254 (1.124–1.399)**
Non-HCPs (*n* = 45,736)	729 (1.59)	1.00	1.00

*AOR* adjusted odds ratio, *CI* confidence interval. ^a^Adjusted for stroke, hypertension, epilepsy, anxiety, depression, and insomnia

**P < 0.001

**Table 3:** Comparison of migraine risks between health care professionals (HCPs) by unconditional logistic regression analysis

	Number (%)	Crude OR (95 % CI)	AOR (95 % CI)^a^
Physicians (*n* = 50,226)	759 (1.51)	0.767 (0.696–0.846)**	1.193 (1.069–1.332)*
Nurses (*n* = 122,357)	4008 (3.28)	1.693 (1.573–1.821)**	1.303 (1.206–1.408)**
Other HCPs (*n* = 45,736)	897 (1.96)	1.00	1.00

*AOR* adjusted odds ratio. ^a^Adjusted for age, gender, stroke, hypertension, epilepsy, anxiety, depression, and insomnia

*P < 0.05; **P < 0.001

**Table 4:** Comparison of migraine risk among physician specialties by unconditional logistic regression analysis

Physician specialists	Number (%)	Crude OR (95 % CI)	AOR (95 % CI)^a^
Internal medicine (*n* = 6110)	79 (1.29)	0.914 (0.706–1.183)	0.973 (0.744–1.271)
Surgery (*n* = 4095)	46 (1.12)	0.793 (0.576–1.090)	0.965 (0.692–1.345)
Obs/Gyn (*n* = 1978)	15 (0.76)	0.533 (0.316–0.901)*	0.550 (0.323–0.937)*
Pediatrics (*n* = 2774)	46 (1.66)	1.177 (0.855–1.620)	1.051 (0.756–1.461)
Emergency medicine (*n* = 479)	5 (1.04)	0.736 (0.302–1.794)	0.681 (0.277–1.672)
Family medicine (*n* = 2568)	53 (2.06)	1.470 (1.087–1.989)*	1.329 (0.973–1.814)
Other specialties (*n* = 15,995)	226 (1.41)	1.00	1.00

*AOR* adjusted odds ratio, *Obs*/*Gyn* obstetrics and gynecology. ^†^Adjusted for age, gender, stroke, hypertension, epilepsy, anxiety, depression, and insomnia

*P < 0.05

Physician specialists had no significant difference in migraine risk than did other physicians, except for Obs/Gyn physician specialists, who had a significantly lower risk (AOR: 0.550; 95 % CI: 0.323–0.937) (Table 4).

## Discussion

This was the first national population-based cohort 5-year study investigating the migraine risk in HCPs. HCPs had a significantly higher migraine risk than did the general population. Among the HCPs, nurses had the highest migraine risk and physicians the second highest; both were significantly higher. Among physician specialties, the only significantly different migraine risk was the lower migraine risk Obs/Gyn specialists.

Previous studies have reported that work stress is related to anxiety, depression, insomnia and hypertension [11, 19–21]. Job strain, a key component of work stress, is a measure of the balance between the psychological demands of a job and the amount of control or decision-making power it affords [11]. In addition to job strain, physical demands, job insecurity, and the amount of support provided by co-workers also play roles in the work stress-illness relationship [11]. Workers with high stress have been shown to have higher rates of a wide variety of diseases than their counterparts with low stress [11]. Our study showed that all three subgroups of HCPs had higher risks of anxiety, hypertension (the difference did not reach statistical significance in nurses), and insomnia than did non-HCPs, which was compatible with previous studies. The risk of depression in HCPs was lower than in non-HCPs. The possible explanations may include the differences in the effects of the different components of work stress encountered by HCPs in Taiwan. Further studies are needed to clarify this issue.

Heavy workloads, work stress, shift work, and sleep disturbance were hypothesized to be the causes of higher migraine risk in HCPs. Working in the hospital was highly stressful because HCPs need to deal with unpredictable medical conditions, have excessive workloads and working hours, are exposed to high levels of stress—especially nurses and physicians, and head nurses and specialist physicians most particularly—and are frequently emotionally exhausted [15]. Comprehensive literature reviews reported that a stress-migraine interaction was hypothesized because of the physiological stress response involved in neuroendocrine, metabolic, and immune changes caused by the activation of the hypothalamic-pituitary-adrenocortical axis, and of the sympathetic nervous system [22–24]. Moreover, stress elicited the onset of migraine, acted as a migraine trigger [25], and could act as a factor of migraine chronification [26].

Shift work and sleep disturbance were also regarded as potential migraine precipitants in HCPs [27, 28]. HCPs, especially nurses, generally work on rotating shifts or night shifts, which might cause sleep problems like difficulty falling to sleep, sleep deprivation, and poor quality sleep [27]. Our study showed that HCPs had a higher likelihood of insomnia than did the general population, which agrees with other studies. According to the epidemiologic data of nurses, working more than 8 night shifts significantly increased the risk of migraine [17]. The association between sleep and migraine was complicated and involved in various models of interaction. Migraine appeared to be associated with the sleep-wake cycle and other circadian biorhythms [29]. The hypothalamus, serotonin, and melatonin are regulators of the pathophysiology of sleep-migraine interaction [29].

The migraine risks for highly stressed physicians specialized in internal medicine, surgery, pediatrics, and emergency medicine were no higher than those for other specialists. Better awareness of migraine, easier access to self-medication, and less time to seek medical care are possible explanations for this finding [30]. However, the finding of a lower migraine risk in obstetricians and gynecologists warrants additional studies to explore the mechanism.

Migraine may cause substantial productivity losses through absenteeism and impaired effectiveness at work [9]. A study about productivity impact of headache on a heavy-manufacturing workforce showed that a small minority (5.7 %) of those with headache, who were only 2.5 % of the workforce, accounted for >45 % of presenteeism-related lost productivity [9]. Therefore, headache disorders are hugely costly to national economies [9].

Our study has some limitations. First, the NHIRD does not provide detailed information about the severity of migraines, scales of stress, number of shifts worked, levels of workloads, patterns of sleep, or other lifestyle and socioeconomic characteristics, which prevented us from investigating the association between these risk factors for migraine. Additional studies on this topic are warranted. Second, this study showed the cumulative incidence rate of migraine during a 5-year follow-up. A longer follow-up, perhaps 10 or 20 years, is undoubtedly needed if we want to clarify the prevalence of migraine in this population. Finally, our study was a nationwide population-based study of a Taiwanese population; however, it might not be generalizable to HCPs in other nations.

## Conclusion

Our findings showed that HCPs in Taiwan had a higher risk of migraine than did the general population. Nurses and physicians were especially vulnerable to migraine. Physician Obs/Gyn specialists had a lower migraine risk than did other physician specialists. Heavy workloads, high work stress, and shift work with sleeping disturbance might be the major precipitating factors of the higher migraine risk in HCPs. These findings not only remind us to raise our awareness of migraine in the healthcare workplace, but also provide important implications for the government and other public health decision makers to set up strategies for dealing with migraine in HCPs.

## Abbreviations

HCPs: healthcare professionals
LHID2000: Longitudinal Health Insurance Database 2000
NHI: National Health Insurance
NHIRD: National Health Insurance Research Database
Obs/Gyn: obstetrics and gynecology
PER: registry of medical personnel

## References

[1] Pryse-Phillips W, Findlay H, Tugwell P, Edmeads J, Murray TJ, Nelson RF (1992). A Canadian population survey on the clinical, epidemiologic and societal impact of migraine and tension-type headache. Can J Neurol Sci.

[2] O’Brien B, Goeree R, Streiner D (1994). Prevalence of migraine headache in Canada: a population-based survey. Int J Epidemiol.

[3] Stewart WF, Lipton RB, Celentano DD, Reed ML (1992). Prevalence of migraine headache in the United States. Relation to age, income, race, and other sociodemographic factors. JAMA.

[4] Lipton RB, Stewart WF, Diamond S, Diamond ML, Reed M (2001). Prevalence and burden of migraine in the United States: data from the American Migraine Study II. Headache.

[5] Lipton RB, Bigal ME, Diamond M, Freitag F, Reed ML, Stewart WF (2007). Migraine prevalence, disease burden, and the need for preventive therapy. Neurology.

[6] Steiner TJ, Birbeck GL, Jensen RH, Katsarava Z, Stovner LJ, Martelletti P (2015). Headache disorders are third cause of disability worldwide. J Headache Pain.

[7] Edmeads J, Findlay H, Tugwell P, Pryse-Phillips W, Nelson RF, Murray TJ (1993). Impact of migraine and tension-type headache on life-style, consulting behaviour, and medication use: a Canadian population survey. Can J Neurol Sci.

[8] Kobak KA, Katzelnick DJ, Sands G, King M, Greist JJ, Dominski M (2005). Prevalence and burden of illness of migraine in managed care patients. J Manag Care Pharm.

[9] Selekler MH, Gökmen G, Steiner TJ (2013). Productivity impact of headache on a heavy-manufacturing workforce in Turkey. J Headache Pain.

[10] WHO (2001). The world health report 2001–mental health: New understanding, New hope.

[11] Wilkins K, Beaudet MP (1998). Work stress and health. Health Rep.

[12] Patrick PK (1979). Burnout: job hazard for health workers. Hospitals.

[13] Ndejjo R, Musinguzi G, Yu X, Buregyeya E, Musoke D, Wang JS, Halage AA, Whalen C, Bazeyo W, Williams P, Ssempebwa J (2015). Occupational health hazards among healthcare workers in Kampala, Uganda. J Environ Public Health.

[14] Lin YW, Chang YW, Tsai CC (2004). Job strain and health-related quality of life of hospital employees: case of a medical center in Taichung. Taiwan J Pub Health.

[15] Wilkins K (2007). Work stress among health care providers. Health Rep.

[16] Lin KC, Huang CC, Wu CC (2007). Association between stress at work and primary headache among nursing staff in Taiwan. Headache.

[17] Wang Y, Xie J, Yang F, Wu S, Wang H, Zhang X, Liu H, Deng X, Yu S (2015). The prevalence of primary headache disorders and their associated factors among nursing staff in North China. J Headache Pain.

[18] National Health Insurance Research Database. Available at: http://nhird.nhri.org.tw/. Accessed December 1, 2015.

[19] Rusli BN, Edimansyah BA, Naing L (2008). Working conditions, self-perceived stress, anxiety, depression and quality of life: a structural equation modelling approach. BMC Public Health.

[20] Akerstedt T, Knutsson A, Westerholm P, Theorell T, Alfredsson L, Kecklund G (2002). Sleep disturbances, work stress and work hours: a cross-sectional study. J Psychosom Res.

[21] Ming EE, Adler GK, Kessler RC, Fogg LF, Matthews KA, Herd JA, Rose RM (2004). Cardiovascular reactivity to work stress predicts subsequent onset of hypertension: the Air Traffic Controller Health Change Study. Psychosom Med.

[22] Chrousos GP, Gold PW (1998). A healthy body in a healthy mind–and vice versa–the damaging power of “uncontrollable” stress. J Clin Endocrinol Metab.

[23] Maier SF (2003). Bi-directional immune-brain communication: implications for understanding stress, pain, and cognition. Brain Behav Immun.

[24] Siniatchkin M, Averkina N, Andrasik F, Stephani U, Gerber WD (2006). Neurophysiological reactivity before a migraine attack. Neurosci Lett.

[25] Chabriat H, Danchot J, Michel P, Joire JE, Henry P (1999). Precipitating factors of headache. A prospective study in a national control-matched survey in migraineurs and nonmigraineurs. Headache.

[26] Scher AI, Stewart WF, Buse D, Krantz DS, Lipton RB (2008). Major life changes before and after the onset of chronic daily headache: a population-based study. Cephalalgia.

[27] Halker R, Vargas B, Dodick D. Sleep, insomnia, and migraine. American Headache Society. Available at: http://www.achenet.org/resources/sleep_insomnia_and_migraine/ Accessed June 14, 2015.

[28] Harnod T, Wang YC, Kao CH (2015). Higher risk of developing a subsequent migraine in adults with nonapnea sleep disorders: a nationwide population-based cohort study. Eur J Intern Med.

[29] Dodick DW, Eross EJ, Parish JM, Silber M (2003). Clinical, anatomical, and physiologic relationship between sleep and headache. Headache.

[30] Chen YT, Huang CC, Weng SF, Hsu CC, Wang JJ, Lin HJ, Su SB, Guo HR, Juan CW (2015). Acute myocardial infarction: a comparison of the risk between physicians and the general population. Biomed Res Int.

